# Dose-Ranging Study of Ramosetron for the Prevention of Nausea and Vomiting after Laparoscopic Gynecological Surgery: A Prospective Randomized Study

**DOI:** 10.3390/jcm8122188

**Published:** 2019-12-11

**Authors:** Jin Sun Cho, Sang Wun Kim, Sugeun Lee, Young Chul Yoo

**Affiliations:** 1 Department of Anesthesiology and Pain Medicine, Anesthesia and Pain Research Institute, Yonsei University College of Medicine, 50-1 Yonsei-ro, Seodaemun-gu, Seoul 03722, Korea; chjs0214@yuhs.ac; 2 Department of Obstetrics and Gynecology, Division of Gynecolgic Oncology, Institute of Women’s Life Science, Yonsei University College of Medicine, 50-1 Yonsei-ro, Seodaemun-gu, Seoul 03722, Korea; SAN1@yuhs.ac; 3 Department of Anesthesiology and Pain Medicine, Yonsei University College of Medicine, 50-1 Yonsei-ro, Seodaemun-gu, Seoul 03722, Korea; EA1788@yuhs.ac

**Keywords:** 5-hydroxytryptamine type 3 receptor antagonist, gynecologic laparoscopy, postoperative nausea and vomiting, ramosetron

## Abstract

Patients undergoing laparoscopic gynecologic surgery and receiving postoperative analgesia with opioids have a high risk of postoperative nausea and vomiting (PONV). We compared the antiemetic efficacy of three doses of ramosetron in this high-risk population. In this prospective, double-blind trial, 174 patients randomly received ramosetron 0.3 mg (R0.3 group; *n =* 58), 0.45 mg (R0.45 group; *n =* 58), or 0.6 mg (R0.6 group; *n =* 58) at the end of surgery. The primary outcome was the incidence of PONV during the first postoperative 48 h. Nausea severity, pain scores, adverse events, and patient satisfaction (1–4; 4, excellent) were assessed. The incidence of PONV was not different between groups (35%, 38%, and 35% in R0.3, R0.45, and R0.6 groups; *p =* 0.905). Nausea severity, pain scores, and incidence of adverse events (dizziness, headache, or sedation) were similar between groups. Compared to the R0.3 group, the R0.45 and R0.6 groups had lower incidence of premature discontinuation of fentanyl-based patient-controlled analgesia primarily because of intractable PONV (9% and 5% vs. 24%; *p =* 0.038), and higher satisfaction scores (3.4 ± 0.8 and 3.3 ± 0.7 vs. 2.4 ± 0.9; *p =* 0.005). Compared to ramosetron 0.3 mg, ramosetron 0.45 and 0.6 mg did not reduce PONV, but reduced premature discontinuation of patient-controlled analgesia and increased patient satisfaction, without increasing adverse events.

## 1. Introduction

Postoperative nausea and vomiting (PONV) is a distressing and common complication of general anesthesia and surgery. Patients undergoing laparoscopic gynecologic surgery and receiving opioid-based patient-controlled analgesia (PCA) after surgery may be at high risk of PONV because of patient and surgical factors [1,2]. The incidence of PONV may be as high as 80% in high-risk patients [3].

Although numerous strategies involving the use of different pharmacological classes (including anticholinergics, antihistamines, phenothiazines, and butyrophenones) have been employed to prevent PONV [4], no approach is universally successful. Currently, 5-hydroxytryptamine (5-HT3) receptor antagonists are recommended as first- and second-line pharmacologic antiemetic agents for PONV prophylaxis because of their efficacy and limited side effects [5]. Ramosetron is the most recently developed selective 5-HT3 receptor antagonist, and is more effective than other 5-HT3 receptor antagonists because of its higher receptor binding capacity and longer half-life [6].

The efficacy of ramosetron 0.3 mg has been established in chemotherapy-induced emesis, and this dose has also been recommended for the prevention of PONV [7,8]. However, the optimal dose of ramosetron has not yet been established in high-risk surgical patients. Although several dose-ranging studies of ramosetron for PONV prophylaxis have been conducted, some did not stratify patients according to surgery- and patient-specific risk levels for PONV [9,10] and others were found to be fabricated and subsequently retracted [11,12]. In addition, little is known about the effective and safe dose of ramosetron that does not produce undesirable adverse effects, such as headache, dizziness, or drowsiness.

We therefore conducted a prospective, randomized, double-blind trial to determine the most appropriate dose of ramosetron for preventing PONV in high-risk patients undergoing laparoscopic gynecologic surgery and receiving opioid-based PCA after surgery.

## 2. Materials and Methods

### 2.1. Study Design and Patients

This prospective, randomized, double-blind study was approved by the institutional review board and hospital research ethics committee of Severance Hospital, Yonsei University Health System, Seoul, South Korea, on 9 February 2015 (#4-2014-1065). It was registered at ClinicalTrials.gov on 17 December 2015 (NCT02478645). Written informed consent was obtained from all participants. Women between 20 and 70 years of age who underwent elective laparoscopic gynecologic surgery from June 2015 to February 2016 were included. All patients were American Society of Anesthesiologists’ (ASA) physical status class I to III. The exclusion criteria were a body mass index > 35 kg·m^−2^, vomiting or receipt of antiemetics or systemic steroids within 24 h before surgery, vestibular dysfunction symptoms, or an allergy to any study drug. Known risk factors for PONV were assessed using the simplified risk scoring system of Apfel et al. [2].

### 2.2. Randomization and Blinding

Using a computer-generated random table, enrolled patients were randomized in a 1:1 ratio to receive 1 of 3 doses of intravenous (IV) ramosetron, regardless of body weight. The doses were 0.3 mg (group R0.3), 0.45 mg (group R0.45), or 0.6 mg (group R0.6). Group assignments were concealed in sealed envelopes. The surgeons, patients, and investigators collecting postoperative data were blind to the group assignment. The anesthesia team and nurses in the post-anesthesia care unit (PACU) and hospital ward were also blind to the group assignment.

### 2.3. Patient Management

Patients fasted before surgery according to local guidelines (at least 8 h for solid foods and 2 h for clear fluids) and received no premedication. Routine ASA monitoring was applied in the operating theater. General anesthesia was induced with propofol 1.5 to 2.5 mg·kg^−1^ and remifentanil 1 μg·kg^−1^, and tracheal intubation was facilitated with rocuronium 0.8 mg·kg^−1^. Anesthesia was maintained with desflurane 5% to 8% in an oxygen-air mixture (FiO_2_ = 0.5) and remifentanil infusion at 0.05 to 0.1 μg·kg^−1^·min^−1^. The desflurane and remifentanil were titrated to maintain the mean blood pressure and heart rate within 25% of baseline values and bispectral index in the range of 40 to 60. At the end of surgery, neuromuscular blockade was reversed with neostigmine 20 μg·kg^−1^ and glycopyrrolate 4 μg·kg^−1^. After emergence from anesthesia, patients received an IV bolus of fentanyl 50 μg for pain control, as well as a single dose of ramosetron (according to group assignment) for PONV prophylaxis. IV-PCA with fentanyl was begun 30 min before the end of surgery for postoperative analgesia. The settings were as follows: basal infusion rate of 0.3 μg·kg^−1^·h^−1^, on-demand 0.15-μg·kg^−1^ boluses, and lockout interval of 15 min. Ondansetron 8 mg was added to the IV-PCA solution (total 100 mL). In addition to this fentanyl-based PCA, all patients received IV ketorolac 30 mg every 8 h for the first 24 h after surgery.

### 2.4. Assessments and Outcome Measures

Postoperatively, the incidence and severity of nausea were assessed during two time periods: 0 to 6 h and 6 to 48 h after surgery. The severity of nausea was evaluated on a five-point numerical rating scale (NRS; 1 = none, 2 = mild, 3 = moderate, 4 = severe, or 5 = intractable). If a patient developed more than moderate nausea or emesis or required an antiemetic, IV metoclopramide 10 mg was administered as a rescue antiemetic. The IV-PCA was halted if severe nausea persisted despite receipt of a rescue antiemetic and/or at the patient’s request. After re-evaluating the nausea level 2 h after IV-PCA was halted, a decision was made to either resume or discontinue the IV-PCA. The number of patients for whom IV-PCA was halted or discontinued was recorded. The level of pain was assessed on an 11-point NRS (0 = no pain to 10 = worst imaginable pain). Patients with breakthrough pain during IV-PCA received additional analgesics upon request or when they reported a pain score ≥ 4. These analgesics consisted of IV fentanyl 50 μg in the PACU and intramuscular (IM) tramadol 50 mg on the hospital ward. Intermittent IM tramadol 50 mg was also administered for breakthrough pain during premature halting or discontinuation of the IV-PCA. We recorded the pain score at 6 h and 48 h after surgery, as well as the worst pain score during 0 to 6 h and 6 to 48 h after surgery. The occurrence of the most common 5-HT3 antagonist side effects—headache, dizziness, and drowsiness—was also recorded. At 48 h after surgery, the patients were asked to rate their overall level of satisfaction regarding analgesia and control of PONV on a four-point scale (1 = poor, 2 = fair, 3 = good, or 4 = excellent).

The primary outcome measure of this study was the incidence of PONV during the first 48 h postoperatively. Secondary outcome measures included the severity of nausea, need for rescue antiemetics, pain scores, additional analgesic requirements, adverse events, early discontinuation of PCA, and patient satisfaction.

### 2.5. Statistical Analysis

Sample size was calculated based on the previously reported 70% incidence of PONV after prophylaxis with ramosetron 0.3 mg in patients undergoing laparoscopic gynecologic surgery [13]. Prior reports defined effective prevention of PONV as a 30% reduction in the incidence of PONV [14]. We therefore estimated that a total of 159 patients would be required to detect a 30% reduction in PONV from a basal incidence of 70%, with 90% power at a significance of *p* < 0.05. We factored in a 10% dropout rate and thereby enrolled 59 patients in each group.

All statistical analyses were performed using SPSS 25.0 (SPSSFW, SPSS Inc., IBM, Armonk, NY, USA) software. After performing the Kolmogorov–Smirnov test for normality of distribution, continuous variables between groups were compared using one-way analysis of variance or Kruskal–Wallis test and expressed as mean ± standard deviation or median (interquartile range). If a statistical difference was noted in multiple comparisons between groups, the *p*-value was adjusted by Bonferroni correction. Dichotomous variables were compared using chi-square or Fisher’s exact tests and expressed as an absolute number (percentage). A *p*-value < 0.05 was considered statistically significant.

## 3. Results

### 3.1. Patients

**Figure 1:**
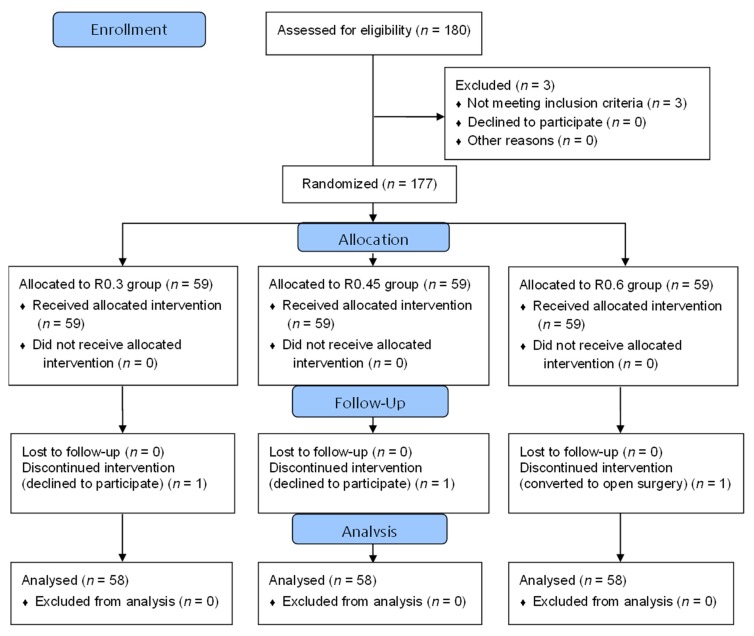
Consort flow diagram.

A total of 177 patients were enrolled and randomized. One patient in the R0.3 group and one patient in the R0.45 group withdrew from the study after randomization because of personal reasons. One patient in the R0.6 group had a large myoma and was excluded from the study when the surgery was converted to an open procedure. Data were analyzed from the remaining 174 patients (58 in each group) (Figure 1). Patient characteristics, including PONV risk factors and operative data, were comparable between the three groups (Table 1).

### 3.2. Postoperative Nausea and Vomiting

The incidence of PONV during the first 48 h after surgery was not statistically different between the three groups (*p* = 0.905): 20 patients (35%) in the R0.3 group, 22 patients (38%) in the R0.45 group, and 20 patients (35%) in the R0.6 group. There were also no significant differences in incidence or severity of nausea, incidence of emesis, or number of patients requiring rescue antiemetics during the first 6 h and 6 to 48 h after surgery between the three groups (*p* > 0.05 for all comparisons) (Table 2).

### 3.3. Postoperative Pain

Postoperative pain scores at 6 h and 48 h after surgery, as well as the worst pain scores during the first 6 h and 6 to 48 h after surgery, were not statistically different between groups. Likewise, the number of patients requiring rescue analgesics was similar between groups (Table 3).

### 3.4. Adverse Events, Premature Discontinuation of IV-PCA, and Patient Satisfaction

The most frequent adverse events were dizziness and headache. There were no statistically significant differences in the incidence of dizziness (*p* = 0.806) or headache between groups (*p =* 0.545). No patient in any group had excessive sedation (Table 4).

The proportion of patients requiring premature discontinuation of IV-PCA was lower in the R0.45 (five patients (9%)) and R0.6 (three patients (5%)) groups than in the R0.3 group (14 patients (24%)) (*p* = 0.005). In the R0.3 group, the reasons for IV-PCA discontinuation were nausea in seven patients, nausea and dizziness in four patients, dizziness in two patients, and headache and dizziness in one patient; in the R0.45 group, the reasons were nausea in four patients and nausea and dizziness in one patient; and in the R0.6 group, the reason was nausea in three patients (Table 4). Patient satisfaction scores regarding control of PONV and analgesia were significantly higher in the R0.45 (3.4 ± 0.8) and R0.6 (3.3 ± 0.7) groups than in the R0.3 group (2.4 ± 0.9) (*p* = 0.005).

## 4. Discussion

In patients undergoing laparoscopic gynecologic surgery and receiving opioid-based analgesia after surgery, ramosetron 0.45 and 0.6 mg did not reduce PONV during the first 48 h postoperatively, when compared to ramosetron 0.3 mg. However, ramosetron 0.45 and 0.6 mg decreased the incidence of premature discontinuation of IV-PCA and increased patient satisfaction.

Among 5-HT3 antagonists, ramosetron has the highest receptor affinity and longest plasma half-life and therefore is the most potent and longest acting [6]. Clinically, ramosetron is more effective for reducing PONV than granisetron [15] or ondansetron [16,17]. In a meta-analysis, ramosetron 0.3 mg was consistently beneficial for preventing PONV (relative risk 0.4, 95% confidence interval (CI) 0.3–0.6), when compared to placebo, and it was also safe, producing no serious adverse events [18]. However, despite prophylaxis with ramosetron, PONV has still been reported in more than 30% of patients [15,17]. The recommended dose of ramosetron for PONV prophylaxis is 0.3 mg, which is the same as for chemotherapy-induced nausea and vomiting, and little is known about the effective and safe dose in high-risk perioperative patients.

The etiology of PONV is multifactorial. Among patient-specific predictors, female sex, previous motion sickness and/or PONV, non-smoking status, and postoperative opioid use have been identified as significant risk factors for PONV (these are the Apfel PONV risk factors) [2]. The type of surgery also significantly influences the occurrence of PONV, with gynecologic (odds ratio (OR) 1.24, 95% CI 1.02–1.52) and laparoscopic (OR 1.37, 95% CI 1.07–1.77) surgery being associated with a higher incidence of PONV, when compared with general surgery [1]. The risk of PONV increased more in patients undergoing both laparoscopic and gynecologic surgeries [19], and up to 66.7% of patients undergoing laparoscopic gynecologic surgery still experienced PONV despite prophylaxis with ondansetron [14]. Ramosetron 0.3 mg showed similar efficacy to ondansetron 8 mg in reducing the incidence of PONV during the first 24 h after gynecologic surgery [8].

Several dose-ranging studies of ramosetron were previously conducted by Fujii et al., but all have been retracted because of data fabrication [11,20,21]. Other studies investigating the effective dose of ramosetron for PONV prophylaxis did not account for surgery- or patient-specific risks. For example, one study investigated the effective dose of ramosetron in patients considered high-risk by Apfel’s classification and reported that ramosetron 0.6 mg was more effective than ramosetron 0.3 mg in reducing PONV during the 6 to 24 h postoperative period [9]. However, that study did not consider the PONV risks associated with patient sex and type of surgery, as both men and women were included and the type of surgery was not differentiated. Female sex is the strongest risk factor for PONV, increasing the risk 2.57 times [4]. In addition, the number of patients in the study (15 or 16 patients per group) was relatively small. These limitations might have contributed to the discrepancy between that study’s results and our findings. Another study compared the efficacy of single-dose (at the end of surgery) and two-dose (at the end of surgery and 4 h later) ramosetron for preventing PONV in patients undergoing laparoscopic gynecologic surgery [10]. Two doses were not superior to one dose. However, the type of anesthesia was not controlled in that study, and three different types were used: propofol induction–volatile anesthetic maintenance, thiopental induction–volatile anesthetic maintenance, and propofol induction–propofol maintenance. Volatile anesthetics are the strongest anesthesia-related predictor and the leading cause of early postoperative vomiting [22,23], whereas propofol reduces PONV, compared with volatile anesthetics [24,25]. In addition, non-opioid based IV-PCA was used for postoperative analgesia in that study, so the risk of PONV may have been lower than in our patients. Thus, the literature contains little reliable data regarding the optimal dose of ramosetron for PONV prevention in high-risk patients. In the current study, most patients had three (71%) or four (26%) Apfel risk factors, with corresponding estimated risks for PONV as high as 61% or 79% [3]. In addition, laparoscopic gynecologic surgery and use of volatile anesthetic agents may have further increased the risk of PONV [19]. Thus, in these highly susceptible patients, we found that increasing the dose of ramosetron to 0.45 and 0.6 mg was not superior to ramosetron 0.3 mg for reducing the incidence of PONV and severity of nausea during the first 48 h postoperatively.

Appropriate postoperative pain relief can facilitate early recovery and increase patient satisfaction. Although opioid-based PCA is a safe and effective method of providing analgesia [26], it also increases the incidence of PONV by up to 50% [27], resulting in early discontinuation of PCA and inadequate pain control. Thus, it is important to identify an adequate regimen that not only reduces pain intensity but also prevents PCA-related PONV. In the present study, higher doses of ramosetron were associated with a reduced incidence of early discontinuation of PCA and increased patient satisfaction regarding postoperative pain relief and PONV. The number of patients in whom IV-PCA was discontinued because of intractable PONV despite rescue antiemetics was much lower in the R0.45 and R0.6 groups than in the R0.3 group. This may have contributed to the higher patient satisfaction scores in the R0.45 and R0.6 groups. Of note, ramosetron 0.45 mg was as effective as 0.6 mg for patient satisfaction.

Although ramosetron has fewer sedative, dystrophic, and extrapyramidal symptoms than antiemetics such as droperidol and metoclopramide, the safety profile of higher ramosetron doses must be considered. Previous studies have reported no differences in the incidence of adverse effects, including dizziness, headache, and drowsiness, with increasing doses of ramosetron [9,10]. Consistent with these findings, our results showed that ramosetron 0.45 mg and 0.6 mg were not associated with increased adverse events compared to ramosetron 0.3 mg.

There are limitations to the present study. First, as we added ondansetron in the IV-PCA solution, we could not exclude the potential impact of ondansetron on PONV. As the incidence of premature discontinuation of IV-PCA containing ondansetron was higher in the R0.3 group, the infused dose of ondansetron was less in the R0.3 group (8, (7.6, 8) mg) compared to the R0.45 (8 (8, 8) mg) and R0.6 (8 (8, 8) mg) groups (*p* = 0.003). However, ramosetron has higher receptor binding capacity and is more effective at reducing PONV than ondansetron [6,16]. When patients who had premature discontinuation of IV-PCA were excluded, the incidence of PONV was not statistically different between the three groups (*p* = 0.246). Second, the observed incidence of PONV in this study was only half of what was assumed in sample size calculation. The higher incidence of PONV in the previous study (70%) [13] might have been attributed to the use of nitrous oxide and younger patient age. We performed multimodal prophylaxis to prevent PONV, including the absence of nitrous oxide, which might have reduced the incidence of PONV. Therefore, there is a possibility that the sample size might be insufficient to detect the assumed effect, with the chance of a type II error. However, when we calculated the effect size using the differences in the PONV incidences between the groups, the effect size was 0.06, which represents a trivial difference in the incidences.

In conclusion, higher doses of ramosetron (0.45 and 0.6 mg) did not reduce PONV in high-risk patients undergoing laparoscopic gynecologic surgery. However, these higher doses reduced premature discontinuation of IV-PCA caused by intractable PONV and increased patient satisfaction, without increasing adverse events, when compared with ramosetron 0.3 mg. Therefore, ramosetron 0.45 mg may be the most appropriate PONV prophylaxis dose for high-risk patients undergoing laparoscopic gynecologic surgery and receiving postoperative opioid-based analgesia.

## Figures and Tables

**Table 1:** Patient characteristics.

	R0.3 Group (*n* = 58)	R0.45 Group (*n* = 58)	R0.6 Group (*n* = 58)	*p*-Value
Age (years)	42.2 (22–64)	41.0 (21–62)	41.2 (20–70)	0.809
Body mass index (kg/m^2^)	23.9 ± 4.2	22.9 ± 3.5	22.8 ± 3.4	0.178
ASA classification (I/II/III)	52/4/2	49/6/3	50/8/2	0.774
Motion sickness (*n*)	14 (24%)	15 (26%)	14 (24%)	0.970
Previous PONV (*n*)	2 (3%)	3 (5%)	0	0.237
Non-smoker (*n*)	56 (97%)	56 (97%)	56 (97%)	>0.999
Apfel’s score				
2 (*n*)	2 (3%)	2 (3%)	2 (3%)	0.996
3 (*n*)	41 (71%)	40 (69%)	42 (72%)	
4 (*n*)	15 (26%)	16 (28%)	14 (25%)	
Operation duration (min)	69.0 (52.0, 100.5)	69.0 (40.0, 104.8)	77.0 (58.5, 111.80)	0.221
Anesthesia duration (min)	95.0 (75.0, 131.3)	95.0 (69.8, 132.5)	100.0 (80.0, 140.0)	0.239
Type of surgery				
Salpingo-oophorectomy	18	15	10	0.585
Ovarian cyst enuclation	15	22	20	
Myomectomy	9	7	10	
Hysterectomy	16	14	18	

Values are mean (ranges), mean ± standard deviation, number (%), or median (IQR). PONV, postoperative nausea and vomiting; R0.3 group, ramosetron 0.3 mg group; R0.45 group, ramosetron 0.45 mg group; R0.6 group, ramosetron 0.6 mg group; ASA classification, American Society of Anesthesiologists’ physical status classification.

**Table 2:** Incidence of nausea and vomiting, and use of rescue antiemetics.

	R0.3 Group (*n* = 58)	R0.45 Group (*n* = 58)	R0.6 Group (*n* = 58)	*p*-Value
**Incidence of nausea**				
Early period (the first 6 h after surgery)	12 (21%)	11 (19%)	12 (21%)	0.965
Late period (6 to 48 h after surgery)	16 (28%)	15 (26%)	14 (24%)	0.914
Overall	20 (35%)	22 (38%)	20 (35%)	0.905
**Severity of nausea**				
Early period (the first 6 h after surgery) (none/mild/moderate/severe/intractable)	46/0/9/2/1	47/1/8/2/0	46/4/7/1/0	0.447
Late period (6 to 48 h after surgery) (none/mild/moderate/severe/intractable)	42/4/4/8/0	43/0/8/7/0	44/1/10/3/0	0.119
Overall	38/2/9/8/1	36/1/12/9/0	38/3/13/4/0	0.666
**Emesis**				
Early period (the first 6 h after surgery)	1 (2%)	2 (3.4%)	3 (5.2%)	0.596
Late period (6 to 48 h after surgery)	0	1 (2%)	2 (3.4%)	0.361
Overall	1 (1.7%)	3 (5.2%)	5 (8.6%)	0.245
**PONV**				
Early period (the first 6 h after surgery)	12 (21%)	11 (19%)	12 (21%)	0.965
Late period (6 to 48 h after surgery)	16 (28%)	15 (26%)	14 (24%)	0.914
Overall	20 (35%)	22 (38%)	20 (35%)	0.905
**Patients requiring additional antiemetics**				
Early period (the first 6 h after surgery)	11 (19%)	10 (17%)	8 (14%)	0.749
Late period (6 to 48 h after surgery)	6 (10%)	13 (22%)	11 (19%)	0.208
Overall	16 (28%)	21 (36%)	16 (28%)	0.507

Values are number (%). PONV, postoperative nausea and vomiting; R0.3 group, ramosetron 0.3 mg group; R0.45 group, ramosetron 0.45 mg group; R0.6 group, ramosetron 0.6 mg group.

**Table 3:** Postoperative pain score and use of rescue analgesics.

	R0.3 Group (*n* = 58)	R0.45 Group (*n* = 58)	R0.6 Group (*n* = 58)	*p*-Value
**Pain score**				
Early period (at 6 h after surgery)	3 (2, 3)	3 (3, 3)	3 (2, 3)	0.229
Late period (at 48 h after surgery)	2 (2, 3)	2 (2, 3)	2 (2, 2)	0.135
**Worst pain score**				
Early period (the first 6 h after surgery)	5 (4, 6)	5 (4, 6)	5 (4, 6)	0.879
Late period (6 to 48 h after surgery)	3 (2, 5)	3 (2, 4)	3 (2, 3)	0.374
**Patients requiring additional analgesics**				
Early period (the first 6 h after surgery)	21 (36%)	17 (29%)	17 (29%)	0.654
Late period (6 to 48 h after surgery)	15 (26%)	14 (24%)	8 (14%)	0.229

Values are mean ± standard deviation or number (%). Pain scores were assessed using an 11-point numerical rating scale (0, no pain, to 10, worst imaginable pain). R0.3 group, ramosetron 0.3 mg group; R0.45 group, ramosetron 0.45 mg group; R0.6 group, ramosetron 0.6 mg group.

**Table 4:** Adverse events.

	R0.3 Group (*n* = 58)	R0.45 Group (*n* = 58)	R0.6 Group (*n* = 58)	*p*-Value
Dizziness	16 (28%)	14 (24%)	13 (22%)	0.806
Headache	10 (17%)	6 (10%)	9 (16%)	0.545
Drowsiness	0	0	0	-

Values are number (%). R0.3 group, ramosetron 0.3 mg group; R0.45 group, ramosetron 0.45 mg group; R0.6 group, ramosetron 0.6 mg group.
